# Matched Behavioral and Neural Adaptations for Low Sound Level Echolocation in a Gleaning Bat, *Antrozous pallidus*

**DOI:** 10.1523/ENEURO.0018-17.2017

**Published:** 2017-03-02

**Authors:** Kevin R. Measor, Brian C. Leavell, Dustin H. Brewton, Jeffrey Rumschlag, Jesse R. Barber, Khaleel A. Razak

**Affiliations:** 1 Graduate Neuroscience Program, University of California, Riverside, CA 92521; 2 Department of Biological Sciences, Boise State University, Boise, ID 83725; 3 Department of Psychology, University of California, Riverside, CA 92521

**Keywords:** active hearing, auditory cortex, bat, echolocation, sensorimotor adaptation, sound level processing

## Abstract

In active sensing, animals make motor adjustments to match sensory inputs to specialized neural circuitry. Here, we describe an active sensing system for sound level processing. The pallid bat uses downward frequency-modulated (FM) sweeps as echolocation calls for general orientation and obstacle avoidance. The bat’s auditory cortex contains a region selective for these FM sweeps (FM sweep-selective region, FMSR). We show that the vast majority of FMSR neurons are sensitive and strongly selective for relatively low levels (30-60 dB SPL). Behavioral testing shows that when a flying bat approaches a target, it reduces output call levels to keep echo levels between ∼30 and 55 dB SPL. Thus, the pallid bat behaviorally matches echo levels to an optimized neural representation of sound levels. FMSR neurons are more selective for sound levels of FM sweeps than tones, suggesting that across-frequency integration enhances level tuning. Level-dependent timing of high-frequency sideband inhibition in the receptive field shapes increased level selectivity for FM sweeps. Together with previous studies, these data indicate that the same receptive field properties shape multiple filters (sweep direction, rate, and level) for FM sweeps, a sound common in multiple vocalizations, including human speech. The matched behavioral and neural adaptations for low-intensity echolocation in the pallid bat will facilitate foraging with reduced probability of acoustic detection by prey.

## Significance Statement

The pallid bat belongs to a small group of “gleaners” that detect prey-generated noise to localize and hunt ground-dwelling prey, while reserving frequency-modulated (FM) sweep echolocation calls for orientation and obstacle avoidance. We show that the bat uses relatively low sound level echolocation calls. Moreover, the bat rapidly adjusts its echolocation calls to keep echo levels at a constant level. Cortical neurons are highly selective for the echo levels that the bat actively maintains with adjustment of vocal output. Thus, the auditory cortex participates in a network that provides context-dependent sensorimotor adjustments of vocal behavior.

## Introduction

Active sensing is the process of adjusting motor patterns to alter sensory inputs (Schroeder et al., 2010). Studies of saccades in primate vision (Rajkai et al., 2008; McFarland et al., 2015), whisking in rodent haptic sense (Pinto et al., 2000; Ritt et al., 2008; Mitchinson and Prescott, 2013), and echolocation in bats (Moss et al., 2011) suggest that active sensing enhances sensory processing by matching inputs to the high resolution analysis provided by specialized neural representations. While studies across sensory systems have primarily described how active sensing enhances spatial or spectral processing, relatively little is known about how signal intensity modifications facilitate sensory processing.

Sound level (intensity) estimation is a fundamental function of the auditory system. Auditory cortex of all species examined shows at least some selectivity for sound levels (bats: Suga and Manabe, 1982; Macias et al., 2014; Hechavarría and Kössl, 2014; cat: Sutter and Schreiner 1995; Phillips et al., 1995; marmoset: Watkins and Barbour, 2011; rat: Phillips and Kelly, 1989; Polley et al., 2004). Quantification of echolocation behaviors across various species of bats shows rapid and context-dependent modifications of call properties, including sound levels (Moss et al., 2011), making bat echolocation a useful behavior to study neural mechanisms of sensorimotor adjustments. Active adjustments of echolocation call levels may enable a bat to maintain constant echo levels during approach to a target. It is hypothesized that such “echo level compensation” allows a matching of sound levels to specialized processing circuits. While a number of studies have examined sound level representations in bat cortex, direct evidence for the neural matching hypothesis requires behavioral and neural measurements in the same species. Here, we tested the neural matching hypothesis in the pallid bat (*Antrozous pallidus*) by measuring the range of sound levels available to the bat under different behavioral conditions and separately recording the degree of neural selectivity for this range.

The pallid bat belongs to a small group of bats (across orders) known as “gleaners.” Gleaning bats listen for prey-generated noise to hunt while reserving echolocation for obstacle avoidance (Denzinger and Schnitzler, 2013). The pallid bat uses echolocation [downward frequency-modulated (FM) sweeps, 60-30 kHz] for obstacle avoidance and listens to prey-generated sounds to hunt terrestrial prey (Bell, 1982). Presumably because echolocation is relatively less important in localizing small prey, gleaners use low sound level echolocation calls (“whispering bats”; Griffin, 1958). Gleaning may have evolved in the context of the use of sounds in predator-prey interactions and suggests strong selective pressure for matched behavioral and neural specializations to process low sound level echolocation calls. Whether gleaning bats perform echo intensity compensation is not known. The neural representation that facilitates low sound level echolocation in gleaning bats is also not known.

The main hypothesis tested in this study is that the pallid bat has matched behavioral and neural adaptations to optimize low sound level echolocation behavior. We first determined whether pallid bats reduced call levels when approaching a target during a food reward task. This experiment was designed to reveal the range of echo levels the pallid bat actively maintains. In addition, to describe the range of possible echo levels available to the bat across target sizes, we broadcast (“ensonified”) recorded pallid bat calls toward wires of various diameters and quantified the returning echo levels. We then recorded level selectivity of cortical neurons specialized for echolocation calls to test whether selectivity matched the range of echo levels documented in the behavioral and ensonification experiments.

FM sweeps are common components of animal vocalizations, including human speech. The mechanisms that generate FM sweep spectrotemporal (direction and rate) selectivity have been characterized (Razak and Fuzessery, 2006, 2008), but the mechanisms that shape level selectivity for FM sweeps remain unclear. Sutter and Loftus (2003) and Wu et al. (2006) suggested level-dependent changes in the timing of interactions between excitatory frequencies and inhibitory sidebands of the receptive field shapes level selectivity for broadband sounds. Here, we tested this model using a two-tone inhibition paradigm to identify mechanisms that shape sound level selectivity for FM sweeps. We show, for the first time, that a gleaning bat performs active echo level compensation to limit the echo levels to a relatively narrow range. The vast majority of neurons in the cortical region selective for the echolocation calls are sensitive and selective for this narrow range of relatively low sound levels. The level-timing relationship between excitatory frequencies and sideband inhibition enhances level tuning for the FM sweeps.

## Materials and Methods

Pallid bats were netted in Arizona, New Mexico, California, and Idaho under each state’s respective scientific collecting permits. The pallid bats at the University of California, Riverside site were housed in an 11 × 14 ft^2^ room. The bats were able to fly in this room and were provided with crickets/mealworms and water ad libitum. The room was maintained on a reversed 12/12 h light/dark cycle. The pallid bats housed at the Boise State University site were cared for following Lollar and Schmidt-French (2002). Housing conditions included a light regime of 10/14 h dark/light. Before behavioral trials, bats were trained to take live mealworms from dishes. All the electrophysiology experiments and the ensonification of wires with pallid bat calls described below were conducted at the University of California, Riverside. The echo level compensation behavior experiment was conducted at Boise State University. All procedures followed the animal welfare guidelines required by the National Institutes of Health and the Institutional Animal Care and Use Committee at the University of California, Riverside and Boise State University.

## Behavioral experiment and ensonification

### Overview

In the behavioral experiment, the task for the bats was to fly to a target, land, and obtain a food reward. The goal of this experiment was to determine whether the pallid bats performed echo level compensation as they approached the target. The calls that the bats produced during this task were then used to ensonify (a method to quantify the information available to bats via echolocation that involves broadcasting ultrasonic bats calls and recording the subsequent echoes) the same target from different distances to estimate the echo levels that the bats received. Next, we conducted an experiment to estimate the range of echo levels the pallid bat may receive from obstacles of different sizes. This approach followed a previous study (Barber et al., 2003) that showed pallid bats are capable of avoiding wires with diameters greater than ∼0.3 mm, but performance approaches chance for smaller wire diameters. Wires of different diameters were ensonified with recorded pallid bat calls to determine the range of echo levels available to bats from these obstacles.

### Methods to determine whether pallid bats performed echo level compensation

The behavioral experiment was performed in a flight room (7.6 × 6.7 × 3 m) lined with sound-attenuating foam. Three pallid bats foraged individually on a dish of live mealworms placed at the end of a cardboard platform (3.6 × 2.4 × 0.2 m). An ultrasonic microphone (Avisoft CM16, ±3 dB, 20-140 kHz) was placed 10.5 cm above the platform and 10 cm behind the center of the dish. It faced the direction of the approaching bat and was angled ∼30° in elevation from the plane of the platform to maximize the duration during which the bat's sonar call was on axis with the microphone during the bat's descent. The microphone was connected via an XLR cable to an Avisoft UltraSoundGate 416H (sample rate = 300 kHz), which connected to a desktop computer running Avisoft Recorder software. Two digital, high-speed infrared-sensitive cameras (Basler Scout,100 frames per second) were oriented at a 45° angle toward each other at the corners of the platform near the mealworm dish, facing the approaching bat. The room was illuminated with Wildlife Engineering infrared LED arrays. The cameras recorded to a desktop computer via a National Instruments PCIe-8235 GigE Vision frame grabber and custom LabView software. Each recording session was triggered with a National Instruments 9402 digital I/O module that synchronized audio and video. A 3D reconstruction of bat flight paths was accomplished by calibrating the flight area using a sparse bundle adjustment algorithm (Theriault et al., 2014) implemented in custom MatLab software (Hedrick, 2008). A fourth-order, low-pass Butterworth filter with a 12 Hz cutoff frequency was used to reduce digitizing error.

To estimate the sound levels of returning echoes, the landing target (bowl of mealworms on the cardboard platform) was ensonified from positions along a transect following a 30° of declination. Sonar calls recorded during three bat approaches (one approach per bat) were played back via an ultrasonic speaker (Avisoft UltraSoundGate Player BL Pro; ±6 dB, 5-110 kHz) while directed at the landing platform and microphone at levels measured during in-flight recordings and from the distances at which the bats emitted the calls. Calls that were closest to emission distances of 25, 37, and 50 cm (three calls per bat, nine total) were selected for playback. To record echoes, a second microphone (Avisoft Bioacoustics CM16; ±3 dB, 20-140 kHz), also connected via an XLR cable to the Avisoft UltraSoundGate 416H (sample rate = 300 kHz), was situated adjacent to the playback speaker. The change in sound level between the target and echo microphones was plotted against distance to obtain logarithmic regressions (dB SPL_rms_: R^2^ = 0.93), which were used to interpolate echo levels of all calls during behavioral trials.

Target and echo microphones were calibrated in dB SPL re 20 μPa using a 40 kHz Reference Signal Generator (Avisoft Bioacoustics) and B&K 2610 measuring amplifier with B&K ¼" microphone type 4939-A-011 (grid on). To obtain dB SPL_rms_ values, the 40 kHz tone was recorded with the same microphones used in the target and echo recordings, while simultaneously measured by the B&K system. A second measurement was taken by the B&K system without the tone to obtain the ambient (mostly low-frequency) room noise levels. The ambient level was then subtracted to obtain the dB SPL_rms_ of the calibration tone. Call levels were measured over each call’s duration and referenced to the calibrated recording in Avisoft SASLab Pro (version 5.2.07). We estimated source dB SPL values at 10 cm from the bat’s mouth by integrating intensities recorded by the target microphone into a geometric spreading formula,

$$L_{1}=L_{2}+20\log_{10}\left(\frac{r_{2}}{r_{1}}\right)$$

where *L* = sound intensity and *r* = distance from the bat’s mouth. Atmospheric attenuation was accounted for following Lawrence and Simmons (1982).

### Methods to determine echo levels produced by wires of different diameters

Wires of 3 different diameters (2.5, 1.2, and 0.3 mm) were individually ensonified using pallid bat echolocation calls played back at different sound levels. The pallid bat easily avoids the 2.5- and 1.2-mm wires while the 0.3-mm wire is near threshold for avoidance (Barber et al., 2003). Each ensonification train consisted of repetitions of a single pallid bat echolocation call, repeated five times at 75 different amplitudes for a total of 375 calls per train. The intercall interval was 100 ms. A single wire of a given diameter with small springs at both ends was suspended vertically from a track on the ceiling and secured to a track on the floor. An ultrasonic speaker (Avisoft UltraSoundGate BL Pro) was mounted on a tripod (height, 1.2 m from the ground) at a distance of 0.5 m from the wire. The ensonification pulses were recorded by an ultrasonic microphone (Sokolich G-II Ultrasonic Probe Microphone System) mounted on a tripod (height, 1.2 m from the ground) at a distance of 0.5 m from the wire plane on the opposite side of the speaker. This microphone directly faced the speaker. Echoes were recorded with a B&K ¼" microphone type 4939-A-011 (grid on). This microphone was mounted adjacent to the speaker, with its transducer in plane with the speaker membrane. Both microphones were calibrated using a 40 kHz Reference Signal Generator (Avisoft Bioacoustics). The tripods were arranged so that the speaker and microphones were aimed directly at the wire.

For each wire diameter, a playlist was created in Avisoft RECORDER USGH, which contained all of the ensonification trains. This playlist was played through the speaker, while recordings from the microphones were made with SpectraPLUS-SC (version 5.2.0.11; Pioneer Hill Software LLC). After completion of the wire conditions, all of the ensonification trains were emitted and recorded with no wire. Each ensonification train was then filtered in Spectra Plus-SC with a high-pass 20 kHz filter. Each train was then analyzed by a custom MATLAB script, which scaled the amplitude to reflect the proper calibration, read the call ID, and then extracted each call and echo pair. Because the call-measuring microphone was 1 m from the speaker, and the echo-measuring microphone was in plane with the speaker (making a total distance of 1 m from the speaker to the wire and back to the microphone), the measurements of the call and echo coincided in time. The call peak was found by simply determining the maximum value within the proper time window, and then the appropriate window was selected within the echo recording, capturing the echo. The script measured the peak-to-peak and Root Mean Square (RMS) level of each call and each echo and then converted these values to dB SPL re 20 μPa. The ambient noise level was subtracted from each call and echo. Linear models were fit to the dataset of each condition.

## *In vivo* electrophysiology

### Surgical procedures

*In vivo* extracellular single unit recordings were obtained from the right auditory cortex of adult pallid bats (both males and females, *n* = 20 bats) anesthetized with isoflurane inhalation, followed by an intraperitoneal injection of pentobarbital sodium (Nembutal, 30 μg/g). To expose the auditory cortex, the head was held in a bite bar, a midline incision was made in the scalp, and the muscles over the dorsal surface of the skull were reflected to the sides. The front of the skull was scraped clean and a layer of glass microbeads applied, followed by a layer of dental cement. The bat was then placed in a custom-made Plexiglas holder. A cylindrical aluminum head pin was inserted through a crossbar over the bat's head and cemented to the previously prepared region of the skull. This pin served to hold the head secure during the recording session. The crossbar holding the head pin was secured behind the bat, leaving no interference between the speaker and the ear. The location of the auditory cortex was determined relative to the rostrocaudal extent of the midsagittal sinus, the distance laterally from the midsagittal sinus, and the location of a prominent lateral blood vessel that lies parallel to the midsagittal sinus (Razak and Fuzessery, 2002). The size of the exposure was ∼2 mm^2^. Exposed muscle was covered with petroleum jelly, and exposed brain surface was covered with silicone oil to prevent desiccation. At the end of the recording session, the incision was sutured, and lidocaine and a topical antibiotic were applied to the wound. The animal was allowed to recover in isolation before the next surgery. Following one to three recording sessions, the bat was euthanized with an overdose of pentobarbital solution and transcardially perfused with 4% paraformaldehyde with subsequent removal of the brain for histologic processing.

### Recording procedures

Experiments were conducted in a warm (∼80°F), sound-attenuated chamber lined with anechoic foam (Gretch-Ken Industries). Bats were kept anesthetized throughout the course of the experiments with additional pentobarbital sodium (one-third of presurgical dose) injections. Acoustic stimulation and data acquisition were driven by custom software (Batlab, Dr. Don Gans, Kent State University) and Microstar DSP board based hardware. Programmable attenuators (PA5, Tucker-Davis Technologies) allowed control of sound intensities before amplification by an integrated amplifier (Yamaha AX430). Stimuli were delivered using an LCY- K100 ribbon tweeter (Madisound) placed 38 cm directly in front of the bat at an elevation aligned with the snout. Because a major goal of this study was to examine level tuning to the FM sweeps used in echolocation, the 0° elevation/azimuth speaker location was chosen as it approximates echoes coming back along the flight path. In addition, most FM sweep-selective neurons tuned between 30 and 60 kHz in the pallid bat auditory cortex are binaurally insensitive (EO/O type neurons) when tested with interaural level differences (Razak and Fuzessery, 2002). Therefore, except for changes in absolute thresholds, the results presented below are likely to be similar at least for a narrow range of azimuths around 0° from which echoes return.

The frequency response curve of the sound delivery system, measured with a 1/4-in microphone (Bruel and Kjaer, type 4939-A-011, grid on) had a roll-off from 30-80 kHz that was gradual at a rate ∼20 dB SPL/octave. The microphone was calibrated with the same 40 kHz calibrated reference signal generator (Avisoft Bioacoustics) used in the wire ensonification experiments. There was no distortion in the measured signals even at the lowest attenuation tested. Sound level (in dB SPL) of the FM sweep used was calculated by finding the area under the frequency-dB SPL calibration curve between the FM sweep endpoints (using a trapezoid rule) and dividing that by the sweep bandwidth. Recordings were obtained using glass electrodes (1 M NaCl, 2-10 MΩ impedance) at depths between 200 and 600 μm. Penetrations were made orthogonal to the surface of the cortex. Action potentials were amplified by a Dagan extracellular preamplifier (2400A) and a spike signal enhancer (FHC) and bandpass filtered (0.3-3 kHz, Krohn-Hite). Extracellular single-unit recordings were identified based on window discriminator threshold crossing and consistency of action potential amplitude and wave form displayed on an oscilloscope. Waveforms and peristimulus time histograms were stored. Responses were quantified as the total number (20 stimulus repetitions, 1 Hz repetition rate) of action potentials occurring within 200 ms of stimulus onset. Adjustments for spontaneous activity were not necessary, because spontaneous activity in these recordings was rare.

### Data acquisition

The focus of this study was on the high-frequency, FM sweep-selective region (FMSR) of the pallid bat A1 (Razak and Fuzessery, 2002). This region is likely to be involved in echolocation behavior. The FMSR contains neurons tuned between 30 and 70 kHz and is located rostral and medial to the lower frequency neurons (tuning 5-35 kHz) that are noise selective (Razak and Fuzessery, 2002). The FM sweep-selective neurons respond better to downward sweeps than to noise or upward sweeps with energy in the same spectral band. To identify the FMSR neurons, tones, noise, and downward FM sweeps that were similar to the natural calls were used as search stimuli. Neurons with characteristic frequency (CF) of >30 kHz and with at least 30% stronger response to downward FM sweeps than noise or upward FM sweeps were isolated for additional analyses. Most FMSR neurons do not respond to noise or upward sweeps (Razak and Fuzessery, 2002).

Once single units were isolated, various response properties were obtained. Pure tones (25–70 kHz, 5- or 10-ms duration, 1-ms rise/fall times, 1 Hz repetition rate) were used to determine the CF and minimum threshold (MT) for tones. CF was defined as the frequency that elicited action potentials to at least four of five successive stimulus repetitions at the lowest level, this level being the MT.

### Level tuning to FM sweeps and CF tones

Downward FM sweeps (5-ms duration, 1-ms rise/fall times, 1 Hz repetition rate) centered approximately at the CF and with bandwidths between 20 and 40 kHz (e.g., 60&cenveo_unknown_entity_wingdings_F0E0;40 kHz or 70&cenveo_unknown_entity_wingdings_F0E0;30 kHz) were presented at different levels (1-5 dB steps increasing from MT). Responses to 20 stimulus presentations were recorded, and a rate level function was plotted (e.g., see Figure 3). The best level (BL) was defined as the level at which the maximum number of spikes was elicited. The dynamic range (DR) was defined as the level range over which spikes count increase from 10% to 90% of maximum response. Two measures were used to quantify level tuning. The percentage turnover (%TO) indicates the monotonicity of the level selectivity function. %TO was calculated as:

$$\mathrm{\%TO}=\frac{\text{Max. Response}-\text{Response at highest intensity}\;}{\text{Max. Response}}\times \text{100\%}$$

(Phillips and Kelly, 1989). Because of the varying values of MT, not all neurons were tested with the same number of levels, so the %TO uses the highest intensity tested or 40 dB above threshold, whichever value is lower. %TO ranges between 0 and 100, with a higher number indicating stronger nonmonotonicity. A level tuning index (LTI) was used to quantify the sharpness of level selectivity as,

$$\left(\frac{\text{N}}{\text{N}-1}\right)\left(1-\frac{\text{mean}}{\text{max}}\right)$$

where *N*, number of levels tested; mean, average response across all tested intensities; and max, maximum response. LTI ranges between 0 and 1, with a higher number indicating sharper level selectivity. In a subset of neurons, level selectivity was determined for both the CF tone and an FM sweep. This allowed an examination of how spectral integration in a broadband and behaviorally relevant sound enhances selectivity compared with pure tones.

### Two-tone inhibition paradigm

To examine the mechanisms of level tuning for FM sweeps, a two-tone inhibition over time (TTI) method was used (Calford and Semple, 1995; Razak and Fuzessery, 2006). Two tones were presented with different delays between them. The frequency of one tone was at the CF (excitatory tone) and was presented at a level of 10-20 dB above threshold and duration of 5-10 ms. The response to the excitatory tone presented alone is noted as the “control response.” The second tone was presented at the same level and with a duration of 5 or 10 ms. The frequency of the second tone was varied between 25 and 70 kHz (1-5 kHz steps), and its onset time was varied with respect to that of the excitatory tone. The center of the range of frequencies that inhibited the response by at least 50% of control response was selected for the remainder of the TTI recordings as the inhibitory tone. In the study, we focused only on the high-frequency inhibitory sideband, because early evidence indicated that the low-frequency sideband was not necessary for level selectivity for downward sweeps. The arrival time of inhibition was defined as the shortest delay between the two tones that produced a 50% reduction of the control response. Negative delays denote that the onset of the excitatory tone occurred before that of the inhibitory tone. Positive delays indicate that the onset of the excitatory tone occurred after that of the inhibitory tone. Inhibition at negative arrival times mean inhibition occurred even when the inhibitory tone was delayed relative to the excitatory tone. Therefore, negative arrival times are interpreted as fast arriving inhibition relative to excitation. Positive arrival times mean inhibition occurred only when the inhibitory tone was advanced relative to the excitatory tone. Positive arrival times are interpreted as slow inhibition relative to excitation. To explore how level affected the arrival time of inhibition, the excitatory and inhibitory tones were covaried in level. Two to five different levels were tested for each neuron based on the MT and usually represented a range of 10-40 dB above thresholds with steps of 5-10 dB. This paradigm allows an investigation of how timing of inhibition relative to excitation changes with increasing sound level.

## Results

The first aim of this study was to determine whether the pallid bats performed echo level compensation during echolocation. The second aim was to perform ensonification experiments with wires to estimate the range of echo levels the pallid bat receives in an obstacle avoidance task. The third aim was to determine whether cortical neurons selective for echolocation calls were level tuned and whether the range of echo levels overlaps with neural tuning. The fourth aim was to identify receptive field mechanisms that lead to level selective neural responses for the echolocation call.

### Pallid bats perform echo level compensation behavior

**Figure 1.**
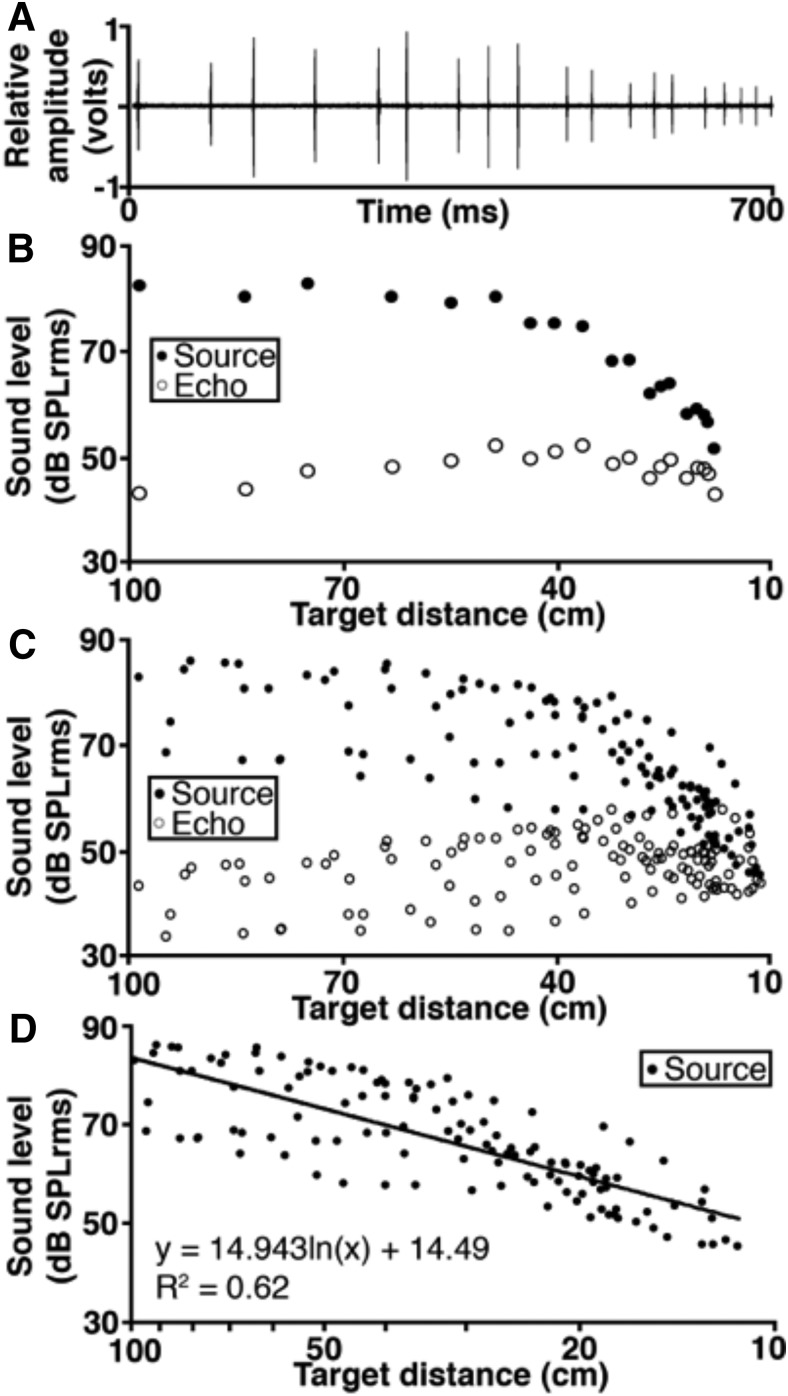
The change in echolocation call levels over time during target approach. ***A***, An oscillogram of call levels produced during a single approach by a pallid bat and recorded by the target microphone beginning at a distance of 100 cm. ***B***, The change in source and echo levels during the same approach as panel A. ***C***, The change in source and echo intensity during trials for three bats (seven total approaches). ***D***, The change in source intensity over time for three bats (seven total approaches). The line represents the logarithmic regression. Source levels decreased on average by 10.4 dB SPLrms per halving of distance.

The bats emitted calls with mean source level of 79 ± 3.2 dB SPL at 1 m from the mealworm target (Table 1). As they approached the landing target, the bats decreased the source levels resulting in the echo levels being maintained in the 30-55 dB SPL range with a mean of 46.8 ± 0.5 dB SPL (Fig. 1, Table 2). The rate of compensation was on average 10.4 dB SPL decrease per halving of distance (Fig. 1*D*). The rates of compensation for bat 1, 2, and 3 were 12.4, 6.8, and 13.3 dB SPL per halving of distance, respectively. The range of compensation rate appears to be correlated with the source level differences (Table 1), suggesting that the louder that the bat calls at 100 cm from the target, the faster the rate of compensation as it approaches the target. These data show that the pallid bat produces relatively quiet echolocation calls and further reduces the call levels to maintain echo levels below ∼55 dB SPL.

**Table 1.** Source levels at 1.0 and 0.5 m from target microphone

Bat(s)	Source level at 1.0 m (dB SPL_rms_)	Source level at 0.5 m (dB SPL_rms_)	*n* (# of approaches)
Bat 1	86.8	81.0	1
Bat 2	70.0 ± 1.2	65.0 ± 2.2	3
Bat 3	85.4 ± 0.8	78.5 ± 1.0	3
Population	79.0 ± 3.2	73.1 ± 3.0	7

Mean source call levels with SEs at 1.0 and 0.5 m from target microphone. Data from individual bats and the population are shown. Due to no bat having called exactly 1.0 nor 0.5 m from the microphone, for each approach, a logarithmic regression formula obtained from calls emitted between 1.0 and 0.3 m was used to estimate the level of a call at 1.0 or 0.5 m from the microphone. The source level at 0.5 m is lower than at 1 m, because the bats have begun to echo intensity compensate (see Figure 1).

**Table 2.** Echo levels of all calls produced within 1.0 and 0.5 m of target microphone

Bat(s)	Echo level within 1.0 m (dB SPL_rms_)	*n* (# of echoes within 1.0 m)	Echo level within 0.5 m (dB SPL_rms_)	*n* (# of echoes within 0.5 m)	# of approaches
Bat 1	52.7 ± 1.3	15	53.7 ± 1.6	11	1
Bat 2	42.1 ± 0.8	44	44.4 ± 0.7	31	3
Bat 3	48.7 ± 0.5	61	49.2 ± 0.6	45	3
Population	46.8 ± 0.5	120	48.0 ± 0.5	87	7

Mean echo levels with SEs of calls produced during approaches of the mealworm target, beginning at 1.0 and 0.5 m from the target. Data from individual bats and the population are shown.

### Echo levels from wires of different diameters

**Figure 2.**
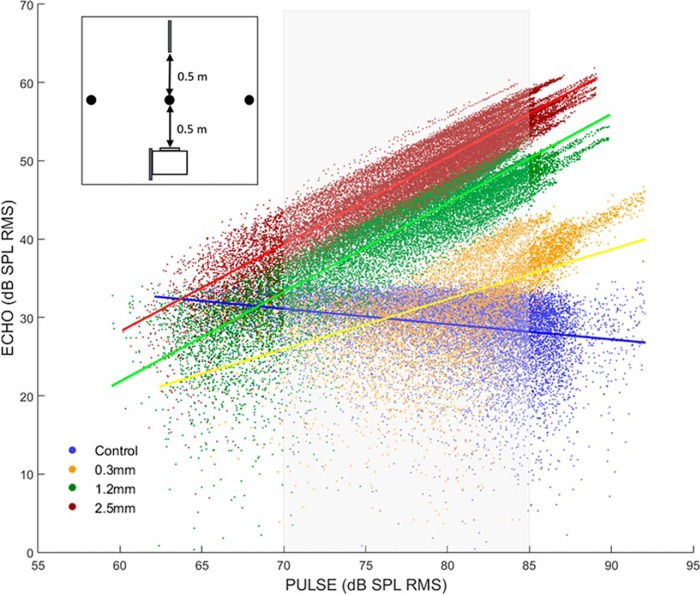
Relationship between pulse and echo levels from ensonification of wires with pallid bat calls. The inset shows a schematic (top-down view) of the ensonification paradigm. A speaker (white rectangle) was used to produce pallid bat calls at various sound levels. The call (pulse) levels were measured with a microphone (thick black line) located 1 m away. Echoes produced by the wire (black circle) were measured with a second microphone (thick black line) located next to the speaker. The main graph shows the echo levels recorded from wires of three different diameters for a corresponding call (pulse) level. The gray shaded rectangle indicates the range of echolocation call levels measured from the bat in the landing task (Table 1).

Here, we measured the range of echo levels at 0.5 m from wires of different diameters by ensonifying the wire with the bat’s own calls. Three different wire diameters were tested. Figure 2 shows the range of echo levels produced for a range of ensonification sound levels. These data show that the echo levels are between 20 and 60 dB SPL when call levels are between 65 and 85 dB SPL (Fig. 2, gray rectangle). The figure highlights the 65-85 dB SPL source range based on the measured source levels in Table 1. In recordings of pallid bat calls made while the bats avoided wires, call levels louder than 85 dB SPL (at a distance of 1 m or less) were rarely observed (data not shown) consistent with the whispering bat moniker. Therefore, we conclude, based on the behavioral and ensonification experiments, that the pallid bat performs echo level compensation behavior to keep echo levels below 55 dB SPL and that the range of echo levels the bat receives from obstacles of different diameters is ∼20-60 dB SPL.

### Neurons in the FMSR are strongly selective for sound levels between 20 and 60 dB SPL

**Figure 3.**
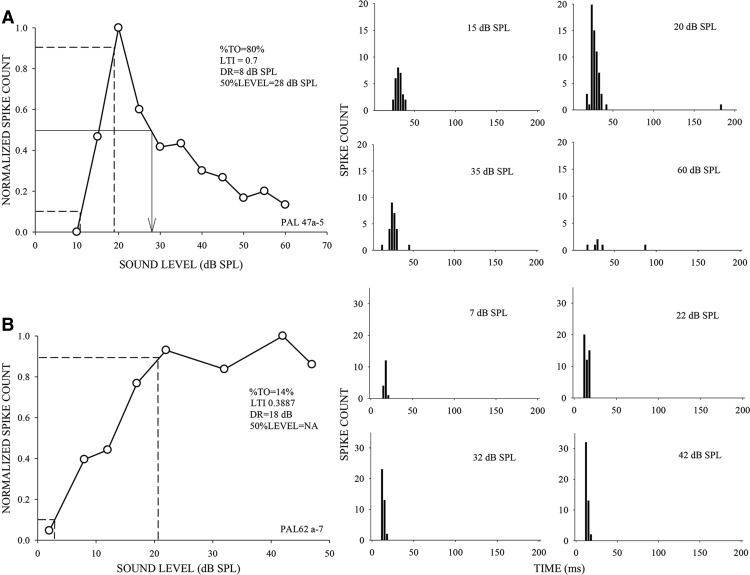
Measures of sound level selectivity. ***A***, Example of a nonmonotonic rate-level response to the sound levels of a downward FM sweep (60-30 kHz, 5-ms duration). Left, Rate-level function of the neuron. The %TO (turnover) is the reduction in response from BL to highest level tested. LTI is a measure of level selectivity (see Materials and Methods for formula). The DR is the range of levels from threshold over which response of the neuron increases from 10% to 90% (marked by dashed lines) of maximum response. The 50%LEVEL (vertical arrow) is the level at which a nonmonotonic neuron’s response declines to 50% of peak response with increasing levels. Right, Post-stimulus time histograms (PSTHs) at selected levels. Stimulus onset is at 0 ms. ***B***, Example of a monotonic neuron. Left, Rate-level function. Right, PSTHs at selected levels. Spike counts were normalized to maximum response for both neurons. The spike count, in this and other relevant figures, is the total number of spikes obtained over 20 repetitions of the stimulus with a recording window of 200 ms from stimulus onset.

**Figure 4.**
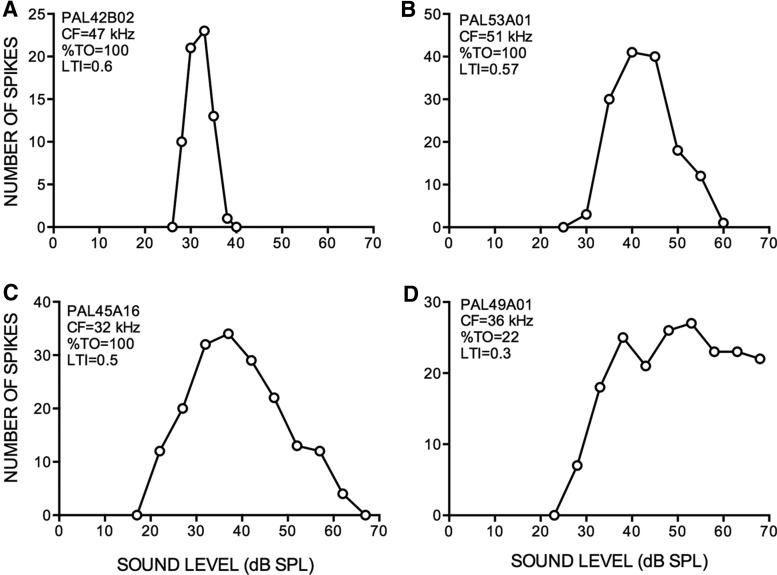
Example rate-level functions recorded from the FMSR to illustrate the range of level selectivity observed.

The rate level functions of 287 neurons from the FMSR of the pallid bat auditory cortex (*n* = 20 bats) were recorded. Unless stated otherwise, downward FM sweeps that were similar in properties (bandwidth and duration) to the pallid bat’s echolocation call were presented as stimuli. The stimuli were presented in the free field at 0° elevation/azimuth in relation to the bat’s snout to mimic echoes along the flight path. Properties of the rate level functions were quantified as described in Figure 3. Figure 4 shows four example neurons to illustrate the range of level selectivity in the FMSR. Neurons in Figures 3*A* and 4*A--C* were classified as nonmonotonic, whereas the neuron in Figures 3*B* and 4*D* was classified as monotonic.

**Figure 5.**
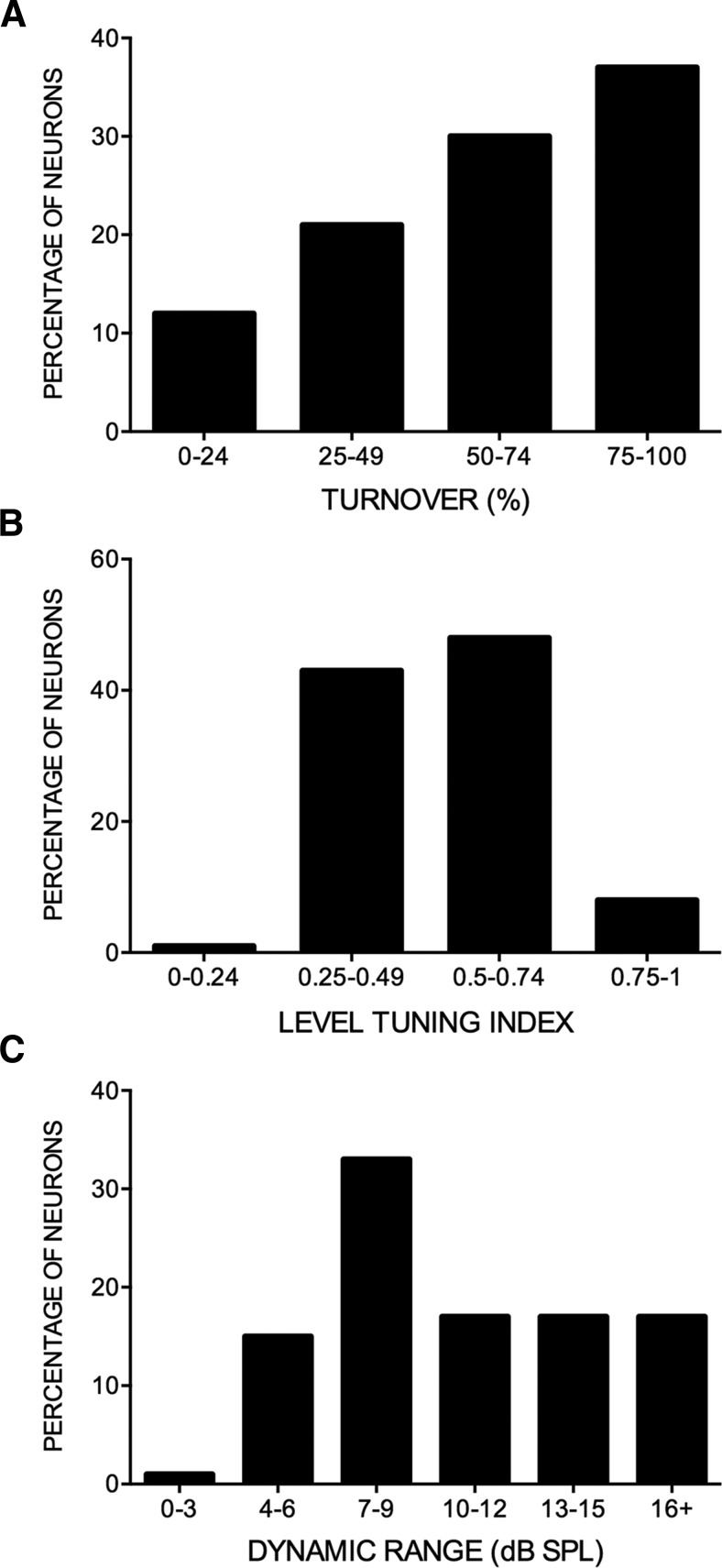
Distribution of sound level selectivity measures in the FMSR of the pallid bat auditory cortex (*n* = 287 neurons). ***A***, %TO distribution from least selective (0-24.9%) to most selective (75-100%). ***B***, Distribution of LTI from least sharply tuned (0-0.2499) to most sharply tuning (0.7500-1.000). ***C***, DR; the range of sound levels over which the response of the neuron increased from 10% to 90% of maximum response. All responses were recorded with downward FM sweeps as stimuli.

Perhaps the most striking feature of the FMSR was the degree of level selectivity seen. For example, the response of the neuron in Figure 4*A* was modulated from maximum to zero (100% TO) with ∼5 dB change in sound level. Figure 5*A* shows the distribution of neurons across four different ranges of %TO values. Sutter and Schreiner (1995) classified neurons with >50% turnover as strongly nonmonotonic. Nearly 70% of FMSR neurons met this criterion (Fig. 5*A*), with ∼40% of these neurons showing %TO of >75%. A further 21% of neurons had %TO between 25% and 50% indicating at least moderate nonmonotonic response (Sutter and Schreiner, 1995). Although the %TO indicates the degree to which the response is modulated between the best and the highest level tested, it does not indicate how level selective a neuron is. For example, neurons in Figure 4*A*,*C* have a 100% TO, but clearly the former is more level selective. Therefore, a LTI was calculated. A neuron that responded to only one of the levels tested will have an LTI = 1; a neuron that responded equally to all the levels tested will have an LTI = 0. The example nonmonotonic neurons in Figure 4*A--C* had an LTI between 0.5 and 0.6. In the FMSR, >50% of the neurons exhibited LTI values >0.5 (Fig. 5*B*), indicating that most FMSR neurons were at least as selective as the example neuron in Figure 4*C*. The DR is a measure of how rapidly (in terms of level) a neuron’s response increases from threshold to maximum. The DR was between 4 and 15 dB in ∼80% of neurons indicating that the BL was near threshold in the vast majority of FMSR neurons (Fig. 5*C*). Taken together, these data indicate that the responses of most FMSR neurons were strongly modulated by stimulus level.

**Figure 6.**
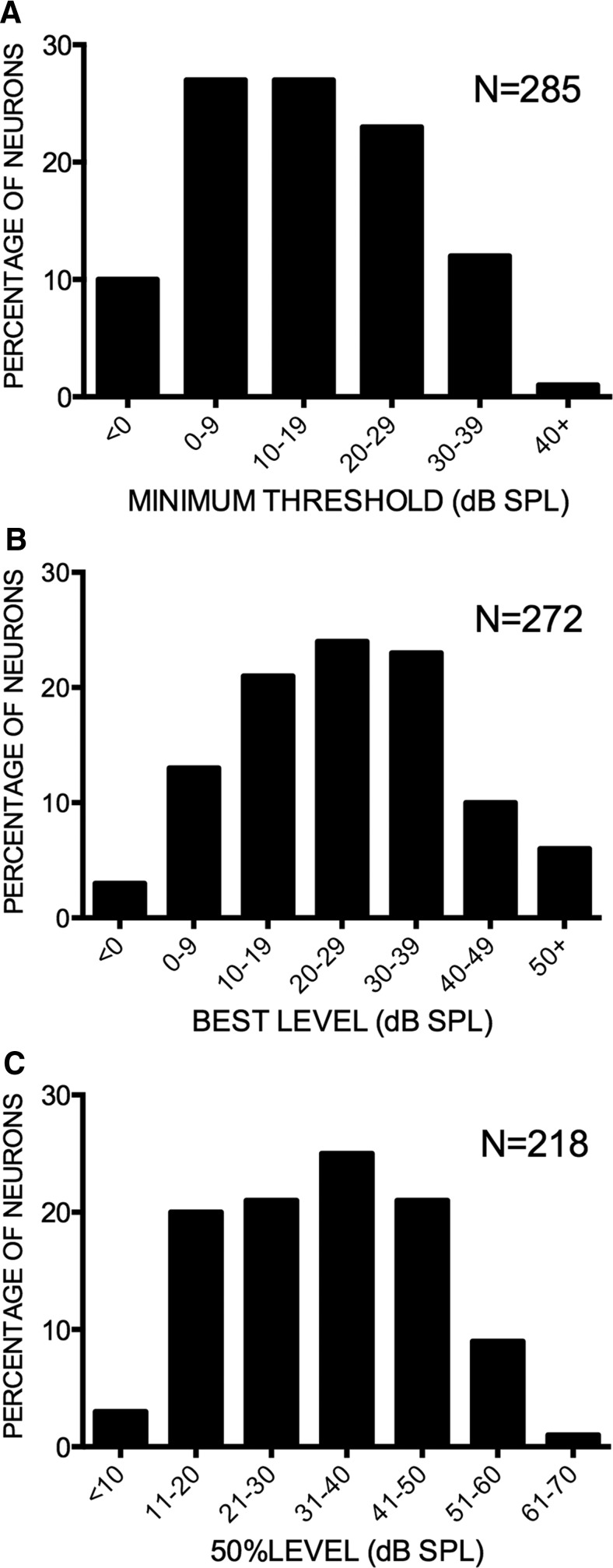
The FMSR is selective for low sound levels. ***A***, Almost all the neurons had a MT < 40 dB SPL. ***B***, The majority of neurons showed BLs between 10 and 50 dB SPL. ***C***, Almost all nonmonotonic neurons with a %TO > 50% had their 50%LEVEL between 11 and 50 dB SPL. The sample size difference between ***A*** and ***B*** is due to the fact that a broad range of sound levels were not tested in some neurons for which MT were obtained.

The FMSR over-represents low sound levels (<50 dB SPL; Fig. 6) providing a substrate to process the relatively quiet echoes the bat appears to receive. The distribution of MTs (Fig. 6*A*) and the BLs (Fig. 6*B*) in the FMSR indicates that the vast majority (∼85%) of FMSR neurons are sensitive to low sound levels and produce peak responses for FM sweeps with levels <50 dB SPL. Almost all the FMSR neurons had thresholds <40 dB SPL. The 50%LEVEL is the center of the range of levels over which the response of nonmonotonic neurons declined to 50% of maximum in the nonmonotonic portion of the rate selectivity function. This measure approximates the center of the range of levels over which the changes in response rate are steep with increasing level. The majority of neurons had 50%LEVEL between 20 and 50 dB SPL (Fig. 6*C*). These data indicate that the FMSR is adapted to process FM sweeps with sound levels that overlaps the range of echo levels that the bat receives (Figs. 1, 2). The high LTI and %TO indicate that the overall population level activity of the FMSR will decrease for echo levels >50 dB SPL. Taken together, the sensitivity and selectivity of FMSR neurons suggest a specialized cortical representation for low sound levels in the echolocation region of the pallid bat auditory cortex.

### FMSR neurons are more selective for levels of FM sweeps than CF tones

**Figure 7.**
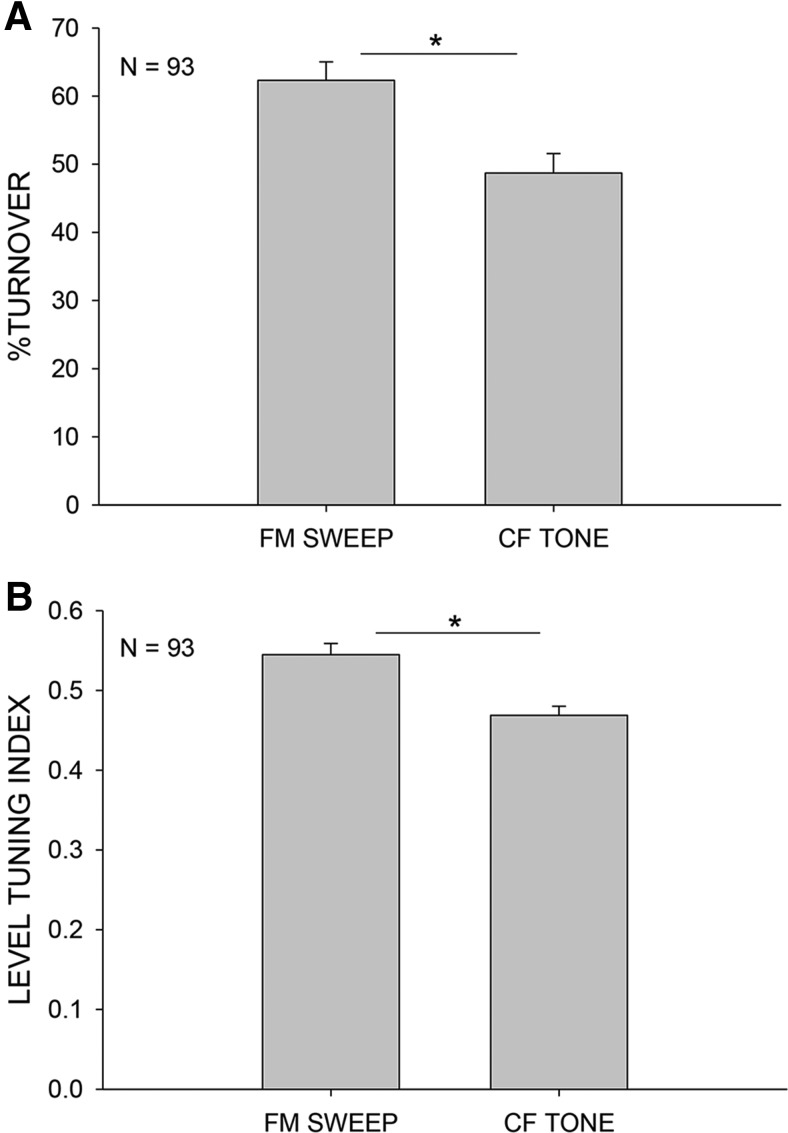
FMSR neurons are more level selective for downward FM sweeps than to the CF tone. These data are from *n* = 93 neurons in which rate level functions were obtained with FM sweep and CF tone. ***A***, Neurons exhibited a higher mean %TO when tested with FM than CF tones (paired *t* test, *p < 0.05). ***B***, The LTI was also significantly higher when tested with the sweep than with CF tone (paired *t* test, *p < 0.05).

Wu et al. (2006) showed using pure tones that the timing and strength of excitatory and inhibitory conductance generated by the same tone can account for nonmonotonic responses. Sutter and Loftus (2003) suggested that integration of excitatory and inhibitory conductance over a broader range of frequencies might increase level selectivity over that obtained from the CF tone alone. The FM sweeps used by the pallid bat for echolocation provides the opportunity to address this question using a behaviorally relevant broadband sound. If across frequency integration enhances level tuning, FMSR neurons should be more level selective for FM sweeps than for CF tones. To test this hypothesis, we compared level selectivity for both CF tone and FM sweeps in a subset of neurons (*n* = 93). On average, FMSR neurons exhibited a significantly higher %TO when tested with downward FM sweeps than with the CF tone (Fig. 7*A*, paired *t* test, *p* < 0.001). Likewise, the average LTI was also higher when tested with downward FM sweeps than CF tone (Fig. 7*B*, paired *t* test, *p* < 0.001). These data suggest that integration across frequencies increases level selectivity in FMSR neurons for the behaviorally relevant downward FM sweep compared with CF tones.

**Figure 8.**
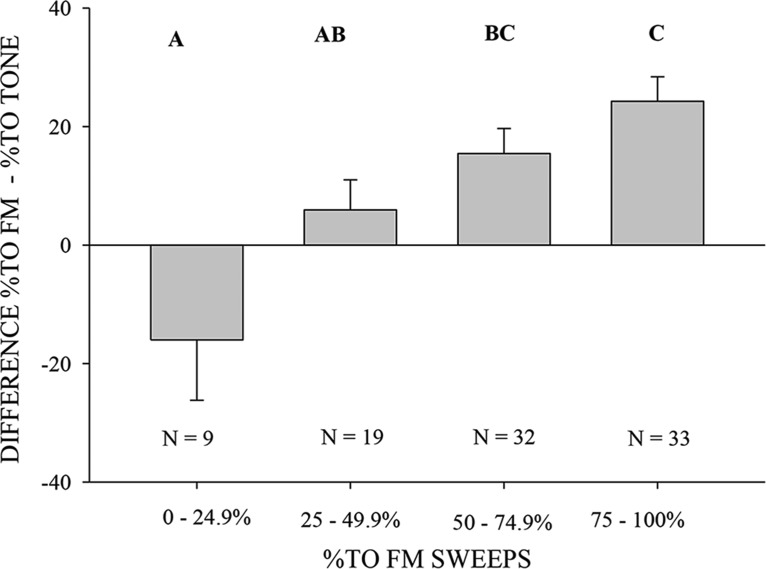
A subset of FMSR neurons is more level selective for FM sweeps than CF tone. Neurons with a high %TO (>50%) for downward FM sweeps have a larger difference in %TO for FM sweeps versus CF tones (one-way ANOVA, *p* < 0.05). Similar letters represent nonsignificant differences (*p* > 0.05) between %TO ranges (Tukey pairwise multiple comparison).

The population average (Fig. 7) does not indicate if, in general, all neurons were more level selective for sweeps over tones, or if there was a subset of neurons with enhanced level selectivity for sweeps compared with CF tone. If the latter were true, it will be possible to focus on these neurons to address the spectrotemporal integration mechanisms. To address this issue, the difference in %TO between FM sweep and CF tone was plotted against the %TO for just the FM sweep (Fig. 8). Neurons that had a high %TO for sweeps had a larger difference in their %TO for the two stimuli. For neurons with the highest %TO for FM sweep (75-100%), the mean difference in %TO for FM and CF tone was 24.3% (±4.1% s.e.); for the 50-75%TO range, the mean difference was 15.4% (±4.2% s.e.); for the 25-50%TO range, the mean difference was 5.9% (±5.1% s.e.); and for the 0-25% range, the mean difference was -16.0% (±10.2% s.e.). A one-way ANOVA showed that there was a significant difference in the mean values among the %TO ranges (*p* < 0.001) and Tukey pairwise comparison showed significant differences in 75-100% versus 0-25% (*p* < 0.001), 75-100% versus 25-50% (*p* = 0.049), and 50-75% versus 0-25% (*p* = 0.005) and no significant differences in 75-100% versus 50-75% (*p* = 0.461), 50-75% versus 0-25% (*p* = 0.530), and 25-50% versus 0-25% (*p* = 0.120). This analysis suggests that, on average, the neurons with %TO > 50% for FM sweep only partially depend on the integration of excitatory and inhibitory conductance generated by the CF tone. Additional contribution to the %TO for FM sweep arises by integrating across a broader range of frequencies. Next, we sought to identify the receptive field mechanism that provides the added level selectivity for the FM sweeps.

### High-frequency sideband inhibition enhances level selectivity for FM sweeps

**Figure 9.**
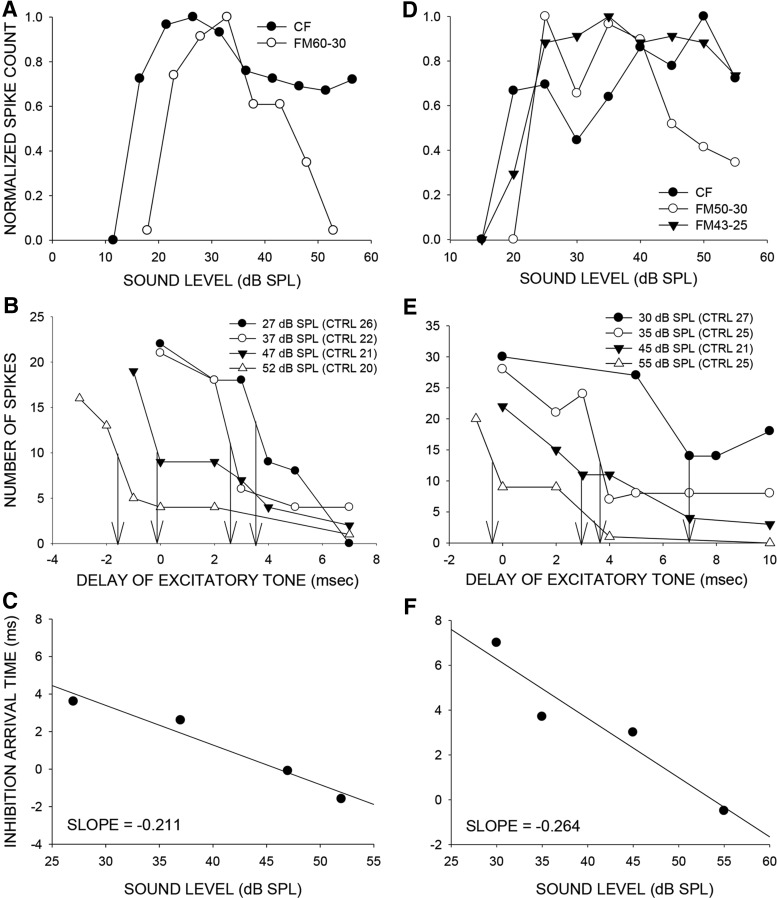
Timing of high-frequency inhibition relative to excitation enhances level selectivity for FM sweep over CF tone. ***A***, Example neuron with a higher %TO for the FM sweep (FM60-30) than CF tone (42 kHz) over the range of levels tested. ***B***, Family of two-tone inhibition curves recorded at different sound levels of the two tones for the same neuron in *A*. The excitatory tone was presented at CF. The numbers in parentheses (CTRL, control) indicate the response to the CF tone presented alone at each sound level. In the two-tone paradigm, the excitatory tone was presented along with an inhibitory tone (46 kHz) from the high-frequency sideband. The delay between the two tones was adjusted as a variable (abscissa) and the response (number of spikes to 20 repetitions of the two tones at a given delay) was measured (ordinate) to generate each curve. Zero delay indicates simultaneous onset, positive delays indicates the excitatory tone was delayed, and negative delays indicates the inhibitory tone was delayed. The level of both tones was changed together as another variable generating the family of four curves. The neural response to the two-tone combination declined below 50% of control excitatory tone alone response (CTRL) at a specific delay (noted as 50% arrival time in the text and marked by the vertical arrows in the figure) in a level-dependent manner. For this neuron, the 50% arrival time systematically becomes faster with increasing stimulus level. For the two highest levels tested, the inhibition in fact occurs even when the inhibitory tone is delayed relative to the excitatory tone indicating that inhibition arrives faster than excitation at these delays. A downward FM sweep presented at these levels will evoke strong inhibition resulting in enhanced level selectivity for FM sweep than CF tone as seen in ***A***. ***C***, A linear regression between the sound level of the two tones versus the 50% arrival time provides the slope that quantifies the rate of change of arrival time with level (-0.211 ms/dB SPL in this neuron). ***D--F***, A second example neuron in which %TO was higher for the FM sweep (FM50-30) than CF (37 kHz) tone. Explanations are similar to the neuron in *A--C*. The inhibitory tone used was 46 kHz. An additional test was conducted in this neuron to determine the necessity of the high-frequency sideband for level selectivity. For this purpose, a downward sweep that started inside the tuning curve (FM43-25) and thereby excluded the high-frequency sideband was used. When the FM43-25 sweep was used, the level selectivity was similar to that obtained with the CF tone, indicating that the high-frequency sideband shapes level selectivity in this neuron.

The nature of across frequency integration was explored to identify mechanisms that enhance level selectivity for FM sweeps over tones. We examined whether the high-frequency sideband was involved in enhancing level selectivity for downward FM sweeps. Using a two-tone inhibition (TTI) paradigm in which the level of the excitatory and the inhibitory tone were increased together, we examined the change in timing of high-frequency inhibition as stimulus level was increased (*n* = 46 neurons). Figure 9*A* shows an example neuron with a higher %TO for the 60-30 kHz downward FM sweep than CF tone (42 kHz). Figure 9*B* shows the TTI plots measured at four different levels. The excitatory tone was at CF. The inhibitory tone used was from the high-frequency sideband (46 kHz). The numbers in legend parentheses indicate the control response (response to excitatory tone alone) at the various levels tested. The response of the neuron was inhibited to 50% of control response when the two tones were presented at 27 dB SPL and with a delay of +3.5 ms (+ indicates that the inhibitory tone was presented first). This delay was noted as the 50% arrival time of inhibition (Razak and Fuzessery, 2006, 2008, 2009). When the level of both tones was increased to 37, 47, and 52 dB, the 50% arrival time decreased to 3, 0, and -2, respectively. The negative arrival time indicates that the neuron will be inhibited even if the excitatory tone onset was before the inhibitory tone. This indicates that the arrival time of inhibition became progressively shorter with increasing level of the two tones (Fig. 9*B*,*C*). The zero and negative arrival times at 47 and 52 dB SPL, respectively, indicate that for a downward sweep at these sound levels, inhibition will always arrive at the neuron before excitation and reduce responses. This is seen as the high %TO at these levels (Fig. 9*A*), thus shaping the stronger nonmonotonic response for the FM sweep compared with the CF tone.

**Figure 10.**
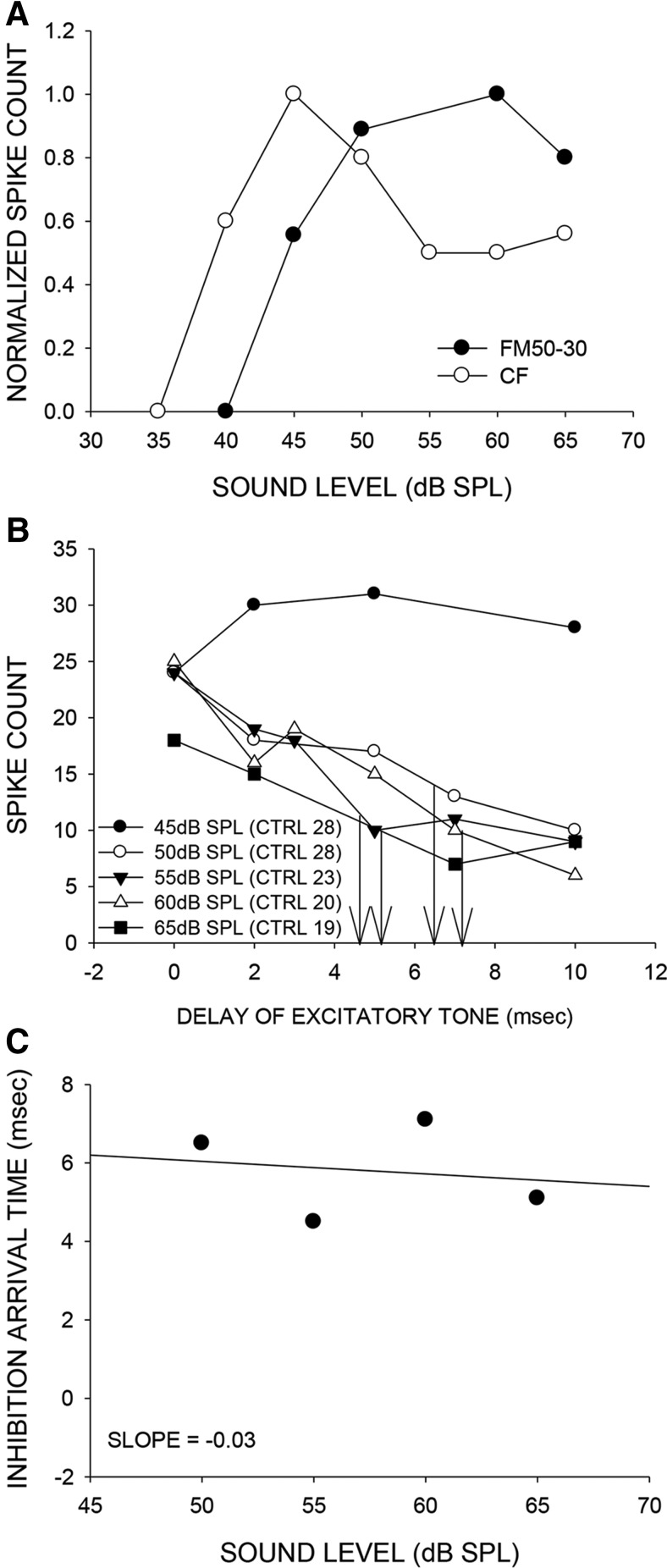
***A***, An example neuron in which the %TO was higher for CF tone (38 kHz) than FM sweep. ***B,C***, The two-tone inhibition family of plots at different tone levels shows that the 50% arrival time does not decrease in a systematic fashion with two tone level. The inhibitory tone used was 43 kHz. The slope of the regression line is ∼0.

Figure 9*D--F* shows a second example in which the neuron was more level selective for a 50-30 kHz FM sweep than the CF tone. All the descriptions for this neuron are the same as for the neuron in Figure 9*A--C*. However, one additional test was performed to determine whether the high-frequency sideband is necessary for enhanced level selectivity. The sweep was started inside the tuning curve (a 43-25 kHz sweep) and thus eliminated the high-frequency sideband from the stimulus (Razak and Fuzessery, 2006). When tested with this FM sweep, the neuron’s level selectivity was similar to that obtained with the CF tone. This indicates that the high-frequency inhibition was necessary to enhance level tuning for a sweep that mimics the natural call. In this neuron as well, TTI performed at multiple sound levels (Fig. 9*E*) showed that arrival time of inhibition became faster as sound levels of both tones were increased. Figure 10 shows a neuron that had the opposite response, in that the %TO was higher for CF tone than FM sweep. The TTI plots show that the arrival time of inhibition did not change in any systematic way with sound level (Fig. 10*E*,*F*). Importantly, although high-frequency sideband inhibition was present in these neurons, the relatively flat inhibition arrival time/sound level relationship correlates with similar sound level selectivity for sweeps and tones. This differs from neurons with inhibition arrival time/sound level curves with large negative slopes (Fig. 9) that correlate with stronger level tuning for FM sweeps than tones.

**Figure 11.**
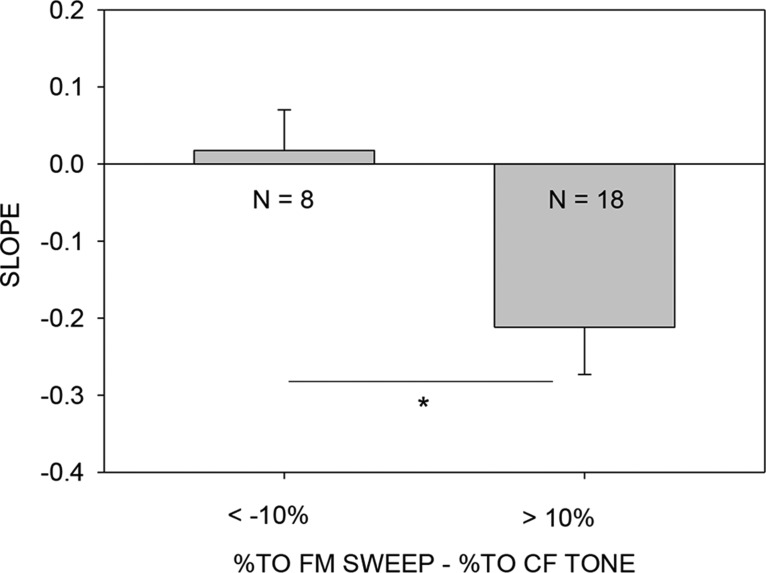
Level-dependent changes in the timing of high-frequency inhibition enhance level selectivity for FM sweeps. Neurons that were more level selective for downward FM sweeps (%TO difference > +10%) than for CF tones had a significantly more negative slope (e.g., Fig. 10) of the regression line between 50% arrival time of inhibition and two-tone level compared with neurons that were more level selective for CF tones than sweeps (%TO difference < -10%) (*t* test, *p* = 0.029).

The relationship between relative timing of high-frequency inhibition and level selectivity exemplified in Figures 9 and 10 held true in the population of 39 neurons in which FM sweep/CF level selectivity and TTI at multiple levels were determined. 18/39 neurons had a %TO for FM sweeps that was at least 10% higher than that for CF tone (e.g., Fig. 9). In 8/39 neurons, %TO for CF tone stimuli was at least 10% larger than it was for FM sweeps (e.g., Fig. 10). A linear regression between arrival time of inhibition and sound level was plotted for these 26/39 neurons (e.g., Figs. 9*C*,*F*, 10*C* ). A negative slope of this regression indicates that the arrival time of inhibition became faster as level increases, while a near zero or positive slope reflects an arrival time that did not change or became slower with increasing level. Figure 11 shows the mean slope of neurons separated according to whether CF tone or FM sweep produced a larger (>10%) turnover with increasing sound level. A *t* test revealed that the slope for the 13 neurons that were more selective for FM sweeps was significantly (*p* = 0.02) more negative than for the eight neurons that were more level selective for CF tones. The remaining 13/39 neurons had similar (within 10%) %TO for CF tone and FM sweep (data not shown). These data indicate that the greater level selectivity that is seen for the FM sweep in some neurons is created by the temporal interactions of the high-frequency sideband inhibition present in the sweep with the excitatory tuning of the neuron. The interaction is level dependent so that an increase in level causes inhibition to arrive earlier and thus reduce the response.

## Discussion

Pallid bats perform echo level compensation to maintain echoes within a relatively constant range (∼30-55 dB SPL; Fig. 1). Most neurons in the FMSR, a cortical region putatively involved in echolocation, are sensitive and selective (Figs. 3-6) for the range of sound levels that matches the range observed in the behavioral and ensonification experiments. Almost all FMSR neurons have thresholds <40 dB SPL and show maximum responsiveness within 15 dB from threshold. Approximately 70% of FMSR neurons exhibit nonmonotonic rate level functions and respond poorly to sounds louder than 50 dB SPL. Neurons provide maximum information along the steepest slope of a response selectivity function (McAlpine et al., 2001; Butts and Goldman, 2006; Yarrow et al., 2012; Yarrow and Seriès, 2015). The slope of level selectivity functions of almost all nonmonotonic neurons (50%LEVEL; Fig. 6*C*) is centered between 20 and 60 dB SPL, thus maximizing information for echo levels measured in the behavioral and ensonification experiments. The preponderance, sensitivity and selectivity of neurons indicate that the FMSR is specialized for a relatively narrow range of low sound levels. Active and rapid sensorimotor compensation matches echo levels to this specialization. These data support the neural matching hypothesis for echo level compensation behavior.

Echo level compensation behavior occurs across bat species that use different types of echolocation calls (Boonman and Jones, 2002; Hiryu et al., 2007; Macías et al., 2016). Aerial-hunting insectivorous bats produce loud (>100 dB SPL) calls to obtain detectable echoes from small prey but reduce call levels when echoes are loud. However, the pallid bat is a gleaner that listens to prey-generated sounds to localize terrestrial prey. Echolocation is used to avoid obstacles along the flight path and for general orientation. Gleaning bats typically emit relatively quiet echolocation calls, and the pallid bat is no exception (∼80 dB SPL at 1 m from source; Tables 1, 2). The present study shows that “whispering” gleaning bats also perform echo level compensation. Both the production and reception components of the echolocation system of the pallid bat have been adapted for low sound levels.

Whispered calls offer stealth against potential sound detection by prey (Miller and Surlykke, 2001; Goerlitz et al., 2010). A disadvantage is that the echoes will be relatively quiet. The low thresholds of most FMSR neurons, and the rapid increase in firing rate at levels near threshold, are likely adaptations to increase sensitivity of FMSR to the low level echolocation system in the pallid bat. Echo level compensation provides the ability to keep functioning at low sound levels even when the reflecting object is close/large. The second potential advantage of the low sound-level echolocation system is as a solution to the clutter problem. If the combined target and clutter echo exceeds the gain of the system, the target might be harder to pull from the noise (Arlettaz et al., 2001). A third reason why the pallid bat may perform echo level compensation and use low level echolocation calls is related to spatial processing. Most FMSR neurons are insensitive to interaural level differences (Razak and Fuzessery, 2002; Razak, 2016). Their spatial tuning is shaped by monaural ear directionality and is sound level dependent (Razak et al., 2015). At low levels, most FMSR neurons are spatially selective to locations along the flight path where echoes are expected. With increasing levels, the spatial receptive fields expand and/or fragment into multiple loci. Therefore, the FMSR may provide accurate spatial information only at low levels due to the spatial resolution offered by narrow receptive fields. At higher levels, the FMSR neurons may serve detection because they are broadly spatially tuned. Thus, by active adjustment of call levels, the bat may use FMSR for both detection and localization.

### High-frequency sideband inhibition enhances level tuning for FM sweeps

Consistent with the model proposed by Sutter and Loftus (2003), temporal interactions between excitatory and sideband inhibitory inputs enhance level tuning in a subset of FMSR neurons. In neurons in which level tuning for CF and FM sweeps were similar, the mechanism proposed by Wu et al. (2006), in which inhibitory conductance generated by the tone begins to precede the excitatory conductance at increasing sound levels, may shape level tuning for CF, with no additional contribution from across frequency integration. For sweeps, inhibition generated by the high-frequency sideband begins to arrive faster than CF excitation at higher sound levels. For the loud downward FM sweeps, high-frequency inhibition will arrive before excitation. Such high-frequency inhibition has sufficient duration (Razak and Fuzessery, 2006) to produce the nonmonotonic rate level responses observed. Iontophoresis studies in the pallid bat indicated that cortical GABAa receptor activity shapes high-frequency sideband of many FMSR neurons (Razak and Fuzessery, 2009). These data suggest that nonmonotonic response to CF tone is shaped subcortically, with further enhancement of selectivity by cortical GABA for broadband sounds. This is consistent with observations in the chinchilla cortex in which GABAa receptor antagonists caused only a few neurons to switch from nonmonotonic to monotonic rate level responses to tones suggesting that CF tone level selectivity in cortex was inherited (Wang et al., 2000). Similar data from the inferior colliculus of the mustached bat indicate that nonmonotonic rate level functions for CF tones may be shaped in the midbrain (Yang et al., 1992). Future studies will determine whether GABAa antagonists have a differential effect on level selectivity for cortical FM sweep versus tone level selectivity as suggested by present data.

### Neural processing constraints on call design

Most bat species use downward FM sweeps to echolocate (Denzinger and Schnitzler, 2013). Laryngeal and expiratory constraints are present at the production level (Suthers and Fattu, 1973, 1982) perhaps making it relatively easier to produce downward FM sweeps of short durations. The present data suggest that an additional constraint may involve neural processing. Spectral, temporal, and level tuning of sideband inhibition in the receptive field predicts direction, rate (Razak and Fuzessery, 2006, 2008, 2009), and level selectivity (this study) for the echolocation call in the pallid bat. The dependence on high-frequency sideband inhibition to shape multiple filters relevant to echolocation along with the differential environmental attenuation of high- versus low-frequency sounds may explain the use of downward FM sweeps in echolocation. Arrival time of high-frequency inhibition is slow relative to excitation in the pallid bat auditory cortex. This allows the neuron to respond selectively to the FM sweeps with fast frequency changes present in the echolocation call. Low-frequency sideband inhibition is faster than excitation, preventing responses to upward FM sweeps (Razak and Fuzessery, 2006). The asymmetry in timing of low (fast)- versus high (slow)-frequency inhibition is thus necessary for shaping multiple filters for a vocalization (FM sweep direction, rate, and level selectivity).

High frequencies are attenuated more strongly over distance than low frequencies. For a downward FM sweep used to echolocate, the returning echoes will contain relatively more energy in the lower frequencies. Neurons tuned in the echolocation frequency range will experience a decreasing gradient of levels from low- to high-frequency portions of their tuning curve. Because the arrival time of inhibition relative to excitation depends on stimulus level (Wu et al., 2006; Razak, 2012; this study), differential environmental attenuation will enhance the asymmetry in the arrival times of low- and high-frequency inhibition generating direction-, rate-, and level-selective responses that match the bat’s calls. If the bat uses upward FM sweeps to echolocate, the attenuation characteristics of the environment will reduce or even cancel the asymmetric sideband timing. Bats use upward FM sweeps in communication calls, but these have longer durations that may depend on different mechanisms to shape selectivity. Thus a combination of production, acoustic, and neural processing constraints may lead to the preferential use of downward FM sweeps to echolocate across bats.

### Methodological issues

Pentobarbital anesthesia was used in the electrophysiological recordings potentially increasing the duration of inhibition in the two-tone inhibition experiments (MacDonald et al., 1989). However, pentobarbital anesthesia is not known to influence arrival time of inhibition. The two-tone inhibition data presented here compares the effect of level on relative arrival time of excitation and inhibition that is likely to be unaffected by anesthesia. While the absolute firing rates may have been affected by anesthesia, the relative measures such as BLs and 50% cutoffs are unlikely to have been affected. Therefore, we interpret the data to indicate a matched behavioral and neural system to enhance processing of low sound level echolocation calls.

### Conclusions

Much like head movements and saccades allow foveal vision to enhance perceptual acuity, we show here that active echo level compensation places echo levels in a specialized cortical region selective for low sound levels. The same receptive field mechanisms that shape spectrotemporal rate and direction selectivity also shape sound level selectivity. An important future line of investigation is whether there is a topographic organization of sound level selectivity and how sound levels are encoded based on distribution of activity across the FMSR neural population. The rapid adjustment of motor output to match sensory inputs to neural specializations in the pallid bat raises intriguing questions about development of sensorimotor integration. It also remains to be seen whether strong level tuning is present before the use of echolocation in flight or whether both echo level compensation behavior and matching level selectivity refine together with echolocation experience. The development of level tuning has not been studied, but Polley et al. (2004) suggest that level selectivity can be enhanced with experience. Future studies will examine concomitant development of behavioral and neural strategies (Sanes and Woolley, 2011) to optimize echo level processing in the pallid bat.

*Note Added in Proof:* The affiliations of 2nd and 5th author was accidentally switched in the version of this article that was published on-line on February 1, 2017, as an Early Release Article. The author and affiliation lines have since been corrected.

## Synthesis

Reviewing Editor: Darcy Kelley, Columbia University Decisions are customarily a result of the Reviewing Editor and the peer reviewers coming together and discussing their recommendations until a consensus is reached. When revisions are invited, a fact-based synthesis statement explaining their decision and outlining what is needed to prepare a revision will be listed below.

The revisions to the manuscript do effectively address the concerns of the original reviewers.

One remaining issue, however, is the term ensonify (ensonification). This somewhat arcane term seems to be defined as “fill with sound”. If so, what does: “Echo levels generated by obstacles of various diameters when ensonified with the bats' calls were between 20-60 dB SPL also overlapping with the range of neural selectivity.”

There must be some way to express what is meant more clearly.
